# Priority setting for improved leukemia management and research in South Africa: a modified Delphi study

**DOI:** 10.1007/s10552-025-01979-4

**Published:** 2025-03-04

**Authors:** Rochelle Woudberg, Edina Sinanovic

**Affiliations:** Health Economics Unit, School of Public Health, University of Cape Town, Cape Town, South Africa

**Keywords:** Leukemia, Priority setting, Delphi method, Consensus, Healthcare professionals, Research priorities

## Abstract

**Purpose:**

In resource-limited environments, setting priorities for leukemia care becomes essential to ensure effective and efficient use of available resources. This study aimed to identify the key areas of leukemia care and services by determining their prioritization within the South African healthcare system and developing a set of improvement and research priorities.

**Methods:**

A two-round modified Delphi method was used to identify leukemia care priorities and rank areas of leukemia management improvement and research priorities. Healthcare professional experts comprised of Clinical Hematologists and Hematopathologists. In round 1, participants independently rated the importance of 125 iterative statements on leukemia care and services derived from literature. In round 2, agreement within the expert participants was considered to finalize the list of priority statements and 17 improvement and research priorities were ranked based on level of importance.

**Results:**

In total, a list of 67 priority statements reached consensus, and 17 improvement and research priorities were established. A high agreement (≥ 90%) was reached for 24 statements within the six themes, these included accurate and advanced diagnostic techniques, factors in determining treatment strategies (e.g., risk stratification), supportive care measures (e.g., pain management and infection prevention), ensuring adequate healthcare workforce, and creating multidisciplinary teams. The highest ranked improvement and research priorities were timely delivery of diagnosis and treatments and biomarker development for early detection, prognosis, and treatment response.

**Conclusion:**

This study identified key priorities for leukemia care within the South African healthcare system, providing an evidence-based framework through expert consensus.

**Supplementary Information:**

The online version contains supplementary material available at 10.1007/s10552-025-01979-4.

## Introduction

Leukemia is a heterogeneous group of blood cancers that are categorized into several distinct subgroups, each with unique biological characteristics and clinical presentations. In 2020, an estimated 474,519 new cases of leukemia were reported worldwide, making it the 15th most commonly diagnosed cancer globally [1, 2].

Additionally, leukemia was the 10th leading cause of cancer-related mortality, with an estimated 311,594 mortalities [1, 2]. According to the Global Burden Disease (GBD) 2021 report, South Africa recorded an estimated 1,786 new leukemia cases and 1,576 leukemia-related deaths, accounting for approximately 2.5% of new cases and 3.1% mortalities worldwide, respectively [1, 3]. Given that South Africa represents about 0.6% of the global population, the proportion of leukemia cases and deaths is disproportionately high relative to its population size. This suggests a higher leukemia burden compared to the global average, potentially due to differences in healthcare access or late-stage diagnoses.

The main priority for cancer is cure, however, this is not always possible. Specifically for people with Leukemia, often the aim is to achieve remission, seek to prolong survival, and ultimately improve quality of life (QoL) [4]. The 5-year survival rate for leukemia patients has substantially increased in recent decades, this improvement has been linked to advances in diagnostic technologies, medical procedures, risk stratification methods, and treatment protocols [5, 6]. Moreover, advances in leukemia treatment methods such as targeted therapy, immunotherapy, and combination protocols in recent years have proven to be more effective than traditional therapies and have fewer side effects improving QoL and ultimately an increase in complete remission (CR) rates [7–9].

Despite substantial progress over the past years, much remains to be done to achieve global equitable access to leukemia care. For example, the 5-year survival rate for leukemia patients have increased up to 70% in the USA, however, the 5-year survival rate has only been reported to be between 10 and 60% in low- and middle-income countries (LMICs) [9–11]. This differential access to various medical, psychosocial, and supportive interventions and good quality leukemia care creates inequity in terms overall survival. Another issue for many countries is the availability of new diagnostic technologies and treatment methods, which are disproportionately unavailable in LMICs. For example, in LMICs these discrepancies are multifactorial, with a high prevalence of comorbidities and infection-related complications. The financial burden on both patients and the healthcare system further limits access to care. Additionally, evidence on optimal management and supportive care is scarce. Many LMICs also face a shortage of skilled healthcare workers, diagnostic resources, and capacity to manage treatment-related side effects [12–16]. Thus, we believe that improving leukemia diagnosis, treatments, and management can reduce mortality, improve treatment outcomes, and increase survival. It can also mitigate negative effects, enhance quality of life, and support progress toward the Sustainable Development Goals (SDGs), particularly good health and wellbeing, and inequality reduction [17].

Currently, there is limited research exploring the priority determinants that are critical to leukemia services, access to cancer care or areas of improvement. Previous South African studies focused on determining access to care for lung cancer [18], or quality indicators for breast cancer [19], however, there is a lack of evidence as to where resources would be best invested for leukemia care in South Africa specifically. Prior studies on priority setting for research [20], development of quality indicators for cancer supportive care [21], and guideline development [22, 23] have used a Delphi-based study to gain consensus to questions or criteria, where evidence is lacking or unavailable. This study aimed to identify the key areas of leukemia care and services by determining their prioritization within the South African healthcare system and developing a set of improvement and research priorities, using a modified Delphi technique for a consensus-based approach.

## Methods

### Study design

The Delphi method is a multi-stage process that uses a systematic approach to obtain group insight toward a current or future challenge and determine the level of consensus by gathering expert-based judgments on a given topic [20, 24]. The process comprises iterative survey rounds whereby controlled feedback on responses are provided and items to be re-rated by participants considering this feedback, thereby allowing reflection on participants own position and an equal opportunity for voice and participation [25].

**Fig. 1:**
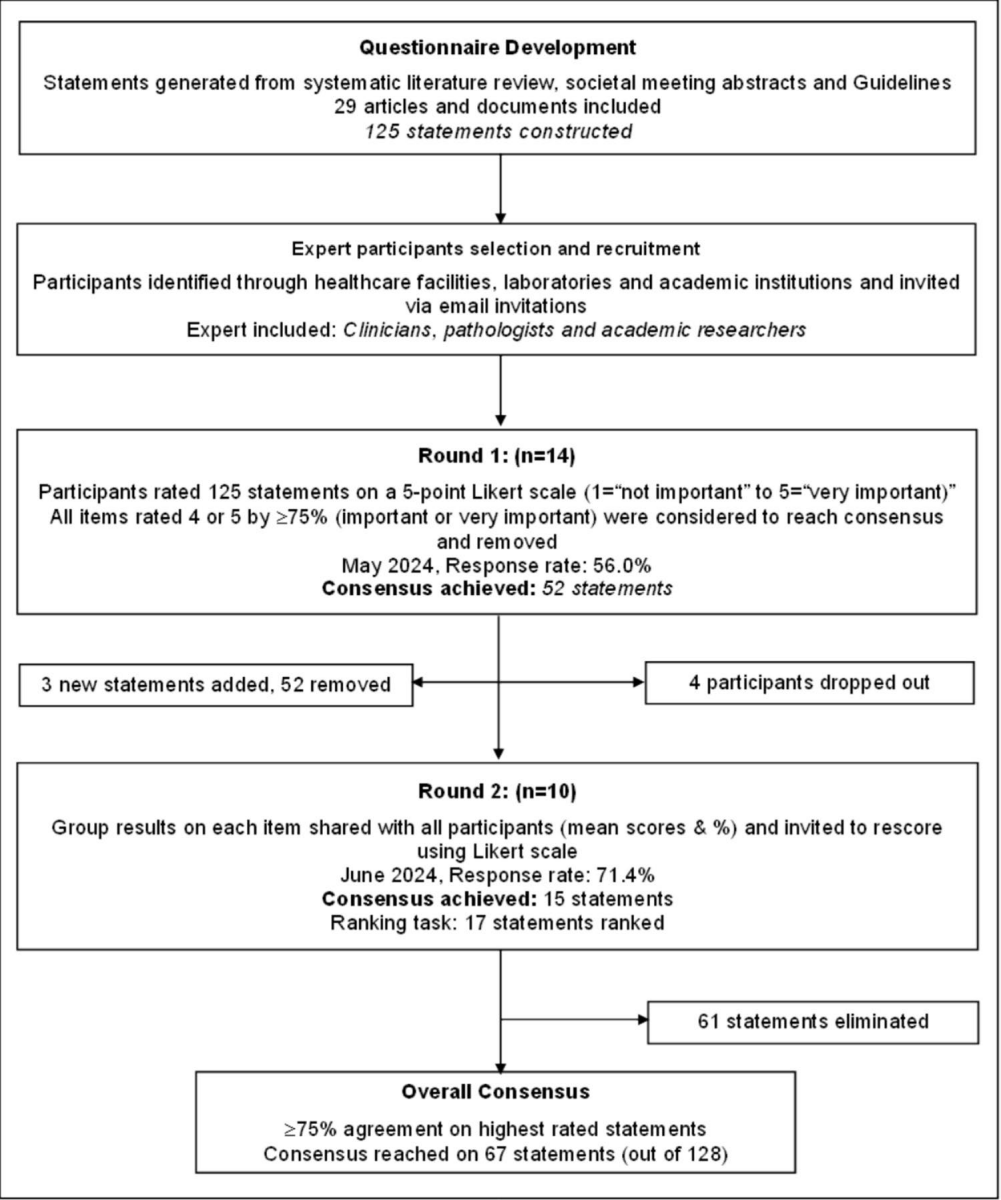
The Delphi process summarized in a flowchart

A structured two-round modified Delphi technique was used to elicit the healthcare providers diverse views and determine relative priorities in leukemia care and services in South Africa. This method was chosen for its flexibility and characteristic features of maintaining anonymity, independent rating of items under consideration, controlled feedback, statistical analysis of group response, and expert input [21, 26, 27]. The study was reported using the Recommendations for the Conducting and Reporting of Delphi Studies (CREDES) guidelines [24]. The process used in this modified Delphi study is presented in Fig. 1.

### Participants

A purposive sampling approach was used to recruit participants to ensure a diverse range of participants in terms of background and experience [28]. Participants selected to ensure representation from different professions, specialist disciplines, and levels of experience and identified from different provinces of South Africa through healthcare facilities, laboratories, and academic institutions.

There has been no agreed-upon sample size in literature for the optimal amount of participant required for a Delphi study. The Delphi study participants and sample size are determined by the study purpose and its limitations; thereby the sample can range from single digits to hundreds [29]. A panel ranging from 7 to 15 participants is usually recommended, based on literature, to ensure sufficient contributions [29–32]. However, the level of expert consensus and quality of data are considered greater importance than the quantity of participants [33].

In this study, experts are referred to as healthcare professionals (HCPs) with substantial experience and knowledge in leukemia care. The HCPs participants primarily consisted of Clinical Hematologists and Hematopathologists, reflecting expertise in both leukemia diagnosis and management. Clinical Hematologists were actively involved in both clinical practice and academic research. Participants were sent an email invitation containing the participant information sheet, access to Round 1, and consent form to complete the Delphi survey. All participants provided electronic informed consent prior to each round and did not receive any compensation for their participation.

### Questionnaire development

The questionnaire was developed for this study (See Supplementary Material: S4). The development of statements used in the Delphi study was informed by a literature review to identify current diagnostic methods, treatments, and management related to leukemia care including meta-analyses and practice guidelines.

A literature search of PubMed and Scopus databases were conducted, and relevant abstracts presented at the meetings of the American Society of Hematology (ASH), European Hematology Association (EHA), and National Institute of Health and Care Excellence (NICE) were reviewed, including the most recent National Comprehensive Cancer Network (NCCN) and European LeukemiaNet (ELN) [34–37].

The search terms included among others “acute leukemia,” “chronic leukemia,” “diagnosis,” “treatment,” “management,” “priority setting,” “priorities,” and “resource allocation.” The search was limited to articles that were published in English only and recent guidelines or articles published in the last 5 years were considered to ensure statements generated reflected the most up-to-date evidence in each area. Overall, 29 articles and documents were included in the questionnaire development. A total of 125 factors were retrieved and translated into statements that formed part of the questionnaire for Round 1 of the Delphi study. The developed statements were conceptually organized into six categorical themes: (1) diagnosis (*n* = 32 statements), (2) treatment (*n* = 50 statements), (3) management (*n* = 26 statements), (4) resource allocation (*n* = 5 statements), (5) access to care (*n* = 7 statements), and (6) quality of life (QoL) (*n* = 5 statements).

### Delphi process

The Delphi study was carried out between May 2024 and June 2024, with each round taking place over a 2-week period. This study involved a two-round online Delphi (Round 1–2) using Microsoft Forms® for all questionnaire rounds. A modified Delphi method was used, where the first round consists of structured closed-ended questions. Modified Delphi methods are frequently used in research to provide list of statements gathered from literature for the questionnaire development to minimize participant burden and reduce the time commitment for experts to take part in the study, therefore reducing the number of rounds required for the study [29, 38]. Furthermore, this approach was selected for this study to ensure participant opinions were gathered on specific factors important for priority setting in Leukemia care specifically.

An invitation email including the participation information sheet and link to the Delphi questionnaire was sent to participants in each round and, where needed, two follow-up reminders were sent. Within the questionnaire link, participants were asked to provide consent, demographic information, and a contact email. The contact emails were used for round 2 questionnaire distribution, as only participants who took part in round 1 were invited to participate in round 2. Participants remained anonymous to one another throughout the study.

The Delphi round 1 questionnaire consisted of 2 sections: (1) participant demographic and (2) rating statements and was available to complete over 2 weeks. Participants in round 1 were asked to provide demographic information and consider their clinical experience to rate the level of importance (priority) of each statement using a 5-point Likert scale (1 = “not important,” 2 = “moderately important,” 3 = “neutral,” 4 = “important,” 5 = “very important”). Participants were also provided with a “no opinion” option if they had no familiarity of the content. A free text field was provided for each section whereby participants could comment on their ratings or provide any additional statements; proposed statements were added if suggested by 2 or more participants.

The Delphi round 2 consisted of 2 sections: (1) rating statement and (2) ranking the priority of a list of improvements and research priority statements. Participants were presented with the results from round 1 in the form of controlled feedback (bar charts plotting group responses to each item, as well as group mean importance rating). The rating of statements followed the same format as round 1, however, statements that reached consensus in round 1 were removed and participants were only asked to rate the importance (priority) of statements that did not reach consensus or were reworked or newly included. Following the rating of statements, participants were asked to rank a list of 10 improvement statements and a list of 7 research priorities.

### Data analysis

Data were de-identified and analyzed using Microsoft Excel® and SPSS® statistical package (version 29.0; IBM, Armonk NY). Participant demographic characteristics were summarized using frequencies and percentages for nominal and categorical variables. Responses were descriptively analyzed and the percentage of each rating, mean score, and standard deviation (SD) for each statement were described to determine the achievement of consensus [39–41].

In this study, consensus was defined by *a priori* criteria of ≥ 75% responses falling within the high-priority categories on the Likert scale (4 = “important” or 5 = “very important”), with ≥ 75– < 90% agreement indicating consensus, and ≥ 90% agreement indicating high consensus, consistent with the previous Delphi studies [28, 41–43]. Statements that were deemed as important by less than 75% of participants were considered as non-consensus and removed after round 2. In the ranking task of round 2, statements were allocated a “weighted score” of 10 (most important) to one (least important) for improvement items and seven (most important) to one (least important) for research priorities. The individual items were weighted and the total scores for each item were calculated to determine the overall priority ranking. Moreover, the Kendall’s Coefficient of Concordance (W) was calculated as a group response agreement of participant’s opinions and evaluated any significant agreement among participants on the ranking task [44].

## Results

### Characteristics of participants

Of the 25 invited experts, 14 completed round 1 (response rate of 14/25, 56.0%), and 10 (response rate of 10/14, 71.4%) completed round 2. The main reasons for non-participation in the Delphi survey rounds were retirement (3), out of office (3), on vacation (1), and the remaining were non-responses (4).

The participant group consisted of 5 (35.7%) Clinical Hematologists in round 1 and 5 (50.0%) in round 2, and 9 (64.3%) Hematopathologists in round 1 and 5 (50.0%) in round 2. Among these participants, 28.6% were males and 71.4% were females, with a majority being in the age range of 36–45 years (57.1%) and > 55 years (28.6%), respectively. Overall, the participants were highly experienced in various areas of expertise and practice settings, providing input from multiple perspectives. The average years of experience was 14.5 (SD 8.09) in round 1 and 15.6 (SD 8.60) in round 2. The demographic and clinical characteristics of participants are presented in Table 1.

**Table 1:** Demographic and clinical characteristics of participants

Characteristics of participants	ROUND 1		ROUND 2	
	Frequency (*n* = 14)	%	Frequency (*n* = 10)	%
Gender				
Male	4	28.6	3	30.0
Female	10	71.4	7	70.0
Age (years)				
25–35	1	7.1	0	0.0
36–45	8	57.1	5	50.0
46–55	1	7.1	1	10.0
> 55	4	28.6	4	40.0
Profession				
Clinical hematologist	5	35.7	5	50.0
Hematopathologist	9	64.3	5	50.0
Areas of Expertise*				
Hematology	9	34.6	6	31.6
Oncology	5	19.2	5	26.3
Acute leukemia	6	23.1	4	21.1
Chronic leukemia	6	23.1	4	21.1
Experience	Mean (SD)		Mean (SD)	
Years of experience in leukemia care	14.5 (8.09)		15.6 (8.60)	

SD = standard deviation

*Multiple answers were permitted

### Delphi process results

The two-round Delphi surveys were conducted between May 2024 and June 2024. In round 1, 125 statements were presented of which 52 reached consensus (≥ 75 agreement) and were removed from the second round. The statements where consensus was not reached (*n* = 73) and three additional statements were re-presented in round 2. In round 2, consensus was reached for 15 statements. A high consensus (≥ 90% agreement) was observed in 24 (37.3%) statements and consensus (≥ 75– < 90% agreement) in 42 (62.7%) statements. A total of 61 statements failed to reach consensus and were eliminated.

Overall, 67 statements reached expert consensus following the Delphi process, these were accepted as priorities for the diagnosis, treatment, and management of leukemia. The statements that reached consensus for agreement, their mean rating, standard deviation, and percentage level of consensus are presented in Table 2.

**Table 2:** Consensus results from the two-round Delphi study, prioritization statements, and their mean rating, SD, and level of consensus

	Round 1 (*n* = 14)		Round 2 (*n* = 10)	
Priority statement	Mean (SD)*	% agreement	Mean (SD)*	% agreement
Diagnosis				
Complete blood counts and Differential counts for the identification of changes in blood components	**5.00 (0.00)**	**100**		
Bone marrow aspiration for assessment of cell morphology	4.86 (0.36)	86		
Cytogenetic analysis for the identification of chromosomal abnormalities associated with specific leukemia subtypes	4.86 (0.36)	86		
Molecular tests (e.g., FISH, PCR, &/or NGS) for the detection of specific genetic abnormalities	**5.00 (0.00)**	**100**		
FISH as an effective technique for molecular testing	4.71 (0.47)	71	**4.70 (0.95)**	**90**
Immunophenotyping for the identification of cell surface markers and antigens	**5.00 (0.00)**	**100**		
Flow cytometry as an effective technique for Immunophenotyping	**4.93 (0.27)**	**93**		
Pregnancy testing in females of childbearing age prior to initiating treatment	4.50 (0.94)	71	4.67 (1.00)	89
Conducting evaluation for testicular involvement in males when indicated	4.43 (0.94)	64	4.60 (0.97)	80
Cardiac function testing to monitor potential treatment-related cardiotoxicity	4.64 (0.84)	79		
Treatment				
Treatment options in Acute Lymphocytic Leukemia:				
Chemotherapy as the primary ALL treatment	4.79 (0.43)	79		
Combination treatment protocol specifically for Philadelphia chromosome-positive ALL	2.64 (2.44)	43	4.60 (0.97)	80
Treatment options in Acute Myeloid Leukemia:				
Chemotherapy as the primary induction AML treatment	**4.93 (0.27)**	**93**		
Stem cell or Bone marrow transplantation in relapse or high-risk AML patients	4.86 (0.36)	86		
Combination treatment protocol for enhanced AML treatment efficacy	2.71 (2.37)	43	4.56 (1.01)	78
Treatment options in Chronic Lymphocytic Leukemia:				
Targeted therapy for enhanced efficacy in CLL	3.79 (1.85)	67	4.50 (1.07)	75
Immunotherapy in relapse or high-risk CLL patients	3.64 (1.91)	64	4.63 (1.06)	88
Treatment options in Chronic Myeloid Leukemia:				
Targeted therapy for enhanced efficacy in CML	**4.93 (0.27)**	**93**		
Factors in determining treatment strategies:				
Age consideration to balance efficacy and tolerance	4.71 (0.83)	86		
Risk stratification for tailoring treatment plans	**4.79 (0.80)**	**93**		
Evaluation of comorbidities to adapt treatment protocols	**4.79 (0.80)**	**93**		
Financial constraints consideration for patients in the public sector	3.93 (1.69)	67	**4.70 (0.95)**	**90**
Measurable residual disease monitoring:				
Flow cytometry in monitoring MRD	**4.93 (0.27)**	**93**		
Use of PCR if available for MRD monitoring	4.71 (0.47)	71	**4.70 (0.95)**	**90**
Additional treatment considerations:				
Adoption of risk stratification in Leukemia treatment	4.71 (0.83)	86		
Combination therapies (e.g., chemo & immunotherapy) for improved treatment outcomes	4.71 (0.83)	86		
Treatment strategies specifically tailored for pediatric leukemia patients	4.71 (0.83)	86		
Use of specific pediatric treatment regimens, for adolescent and young adult patients	**4.79 (0.80)**	**93**		
Utilizing patient MRD status for treatment decisions	4.79 (0.43)	79		
Development of standardized treatment guidelines for the different types of leukemia	**4.93 (0.27)**	**93**		
Education for Healthcare Professionals about emerging treatment paradigms and guidelines	**4.93 (0.27)**	**93**		
Management				
Follow-up & monitoring:				
Routine blood tests for monitoring patients’ response to treatment and detecting early signs of relapse	4.57 (1.09)	85		
PROs in follow-up visits to assess symptoms, quality of life, and treatment-related adverse effects	4.71 (0.61)	79		
Minimizing treatment-related toxicities and long-term adverse effects	4.71 (0.61)	79		
Developing early intervention strategies for managing treatment-related complications	4.71 (0.61)	79		
Guidelines for treatment-related complications and post-treatment follow-up	**4.79 (0.58)**	**93**		
Symptoms and Adverse effects:				
Hematological symptoms (e.g., anemia, thrombocytopenia) management to improve treatment tolerance and prevent complications	**4.93 (0.27)**	**93**		
Nausea and vomiting management to improve treatment adherence	4.64 (0.63)	71	4.60 (0.97)	80
Treatment of mucositis and oral complications for effective pain management	4.71 (0.61)	79		
Supportive Care Measures:				
Pain management to improve quality of life	**5.00 (0.00)**	**100**		
Nutritional support for an improved treatment response and preservation of functional status	4.79 (0.43)	79		
Psychological support to improve overall patient management	**4.93 (0.27)**	**93**		
Blood transfusions as a supportive care measure to improve patient tolerance to aggressive therapies	4.86 (0.36)	86		
Infection prevention to reduce treatment-related morbidity	**4.93 (0.27)**	**93**		
Patient education:				
Providing educational materials for newly diagnosed leukemia patients about their diagnosis and treatment options	4.71 (0.83)	86		
Education about potential adverse effects and given clear instructions for call parameters for any toxicities to treatments	4.86 (0.36)	86		
Educating leukemia survivors about the signs and symptoms of disease recurrence	4.86 (0.36)	86		
Providing education material and communications that meet diverse needs and preferences of leukemia patients (e.g., language, literacy level, cultural background)	4.71 (0.47)	71	4.56 (1.01)	78
Resource allocation				
Availability of affordable and accessible basic diagnostic technologies	4.86 (0.36)	86		
Availability of standard chemotherapy for different types of leukemia	4.86 (0.36)	86		
Availability of diagnostic infrastructure for specialized tests (e.g., flow cytometry, genetic analysis, molecular testing)	4.86 (0.36)	86		
Availability of resources to manage treatment-related adverse events	**4.93 (0.27)**	**93**		
Improving healthcare infrastructure in underserved or remote areas	4.86 (0.36)	86		
Access to care				
Ensuring equitable access to specialist diagnostic resources, services, equipment, and supplies (e.g., imaging, hematology, pathology, chemotherapy, radiation oncology)	4.79 (0.43)	79		
Timely delivery of diagnosis and treatments	**4.79 (0.27)**	**93**		
Adequate healthcare workforce and service providers	**5.00 (0.00)**	**100**		
Multidisciplinary teams in oncology therapy sites (e.g., hematologists, clinical nurses, clinical pharmacists, laboratory specialists)	**4.57 (1.34)**	**92**		
Adequate guidelines on patient referrals and management	4.86 (036)	86		
Equitable access to novel and costly leukemia treatment options	4.43 (1.34)	71	4.60 (0.97)	80
Access to follow-up care and monitoring services, including those in underserved or remote areas	4.86 (0.36)	86		
*Building relationships with staff in peripheral hospitals to improve the overall quality of care	N/A	N/A	4.60 (0.97)	80
Considering long-term effects and survivorship issues when prioritizing leukemia treatment approaches	4.71 (0.47)	71	4.70 (0.95)	**90**
Providing supportive care interventions to improve patient outcomes and overall quality of life	4.79 (0.43)	79		
Addressing emotional well-being (e.g., anxiety, depression) as part of patient care	4.79 (0.43)	79		
Assessing potential financial burden in treatment scheduling and options for patients	4.71 (0.47)	71	4.70 (0.95)	**90**
Early integration of palliative care services in leukemia treatment pathway	4.86 (036)	86		
Kendall’s Coefficient (W)	W	*P*	W	*P*
All statements	0.411	< 0.001	0.465	< 0.001

Factors that have reached high consensus (rating of ≥ 90%) are shown in **bold**

*SD* standard deviation, *ALL* Acute Lymphocytic Leukemia, *AML* Acute Myeloid Leukemia, *CLL* Chronic Lymphocytic Leukemia, *CML* Chronic Myeloid Leukemia, *FISH* Fluorescence in situ hybridization, *MRD* Measurable residual disease, *NGS* Next-generation sequencing, *PCR* Polymerase chain reaction, *PROs* Patient-reported outcomes, *QoL* Quality of life

*Additional statements suggested by participants in round 1

### Priority statements

The 67 (54%) statements that reached consensus as a priority covered a range of topics within each theme (Table 2). Among these, 24 statements received high agreement, including five statements in diagnosis, ten in treatment, five in management, one in resource allocation, and three in access to care. Several statements were related to diagnostic techniques (e.g., molecular testing for the detection of specific genetic abnormalities, Immunophenotyping for the identification of cell surface markers and antigens, and flow cytometry for the technique of choice for immunophenotyping and FISH for molecular testing). These diagnostic strategies are seen as critical for accurate classification and prognosis in leukemia patients.

Factors to determine in treatment strategies (e.g., risk stratification and comorbidities) and measurable residual disease (MRD) monitoring (e.g., flow cytometry and PCR if available) were identified for treatment. Follow-up monitoring (e.g., guidelines for treatment complications), symptoms and adverse effects (e.g., hematological symptoms management), and supportive care measures (e.g., pain management, psychological support, and infection prevention), also received high priority. Lastly, resource allocation (e.g., availability of resources for treatment of adverse events) and access to care (e.g., timely delivery of diagnosis and treatments, adequate workforce, and multidisciplinary teams) were deemed high priority.

The top five priorities identified were diagnostic techniques and accessibility, treatment planning and risk stratification, follow-up monitoring and symptom management, multidisciplinary teams and access to care, and the role of biobanks. These priorities highlight key gaps in leukemia care, including challenges in access to specialized treatment, the need for standardized diagnostic protocols, and research opportunities tailored to the South African context. Each priority was assessed for feasibility, impact, and alignment with existing healthcare policies. From the comments it was determined that participants based their opinions on the current availability and accessibility of items related to the South African healthcare setting. In addition, the Kendall’s coefficients of concordance (W) in the two rounds were 0.411 and 0.465, respectively, which were both statistically significant. Thus, the ratings of participants were consistent, and there was no requirement to conduct a third round of the Delphi survey.

### Ranking priorities

The results of the weighted score, priorities in rank of order from most to least important of the ranking task, and Kendall’s coefficients of concordance (W) for improvement indicators are presented in Table 3 and research priorities are presented in Table 4. The highest ranked statements on priority indicators for improvements suggested the improvement of cancer services in order to provide timely diagnosis and treatment, development of standardize treatment guidelines, recruitment and retention of adequate workforce, and implementation of multidisciplinary teams.

**Table 3:** Improvement prioritization statements, weighted score, and overall rank

Improvement priority items	Weighted score	Rank
Improving timely delivery of diagnosis and treatments	93	1
Development of standardized treatment guidelines for different types of leukemia	79	2
Ensuring adequate healthcare workforce and service providers	77	3
Implementing multidisciplinary teams in oncology therapy sites (e.g., hematologists, clinical nurses, clinical pharmacists, laboratory specialists)	76	4
Improving access to effective but expensive treatment options	62	5
Improving healthcare infrastructure in underserved or remote areas	49	6
Providing supportive care interventions to improve patient outcomes and overall quality of life	47	7
Educating leukemia survivors about the signs and symptoms of disease recurrence	33	8
Developing community health programs to raise awareness and provide early detection services in underserved areas	22	9
Establishing biobanks for leukemia in South African	12	10
Kendall’s Coefficient (W)	0.790 (*P* < 0.001)	

Kendall’s Coefficient of Concordance (W) measures expert agreement across multiple statements. Statistically significant agreement was measured using p < 0.001

**Table 4:** Research prioritization statements, weighted score, and overall rank

Research priority items	Weighted Score	Rank
Investment in the identification and development of biomarkers for early leukemia detection, prognosis, and treatment response	56	1
Exploring long-term outcomes and quality of life for leukemia survivors to inform supportive care strategies	43	2
Research and development of novel therapies, including immunotherapy and targeted therapy, for leukemia in South African setting	42	3
Studying factors affecting treatment adherence and developing interventions to improve compliance	41	4
Evaluating the cost-effectiveness of novel therapies compared to traditional chemotherapy to inform treatment decisions and policy-making	35	5
Researching mechanisms of drug resistance in leukemia to identify strategies to overcome resistance	33	6
Creating personalized risk stratification models based on genetic, molecular, and clinical data	30	7
Kendall’s Coefficient (W)	0.439 (*P* < 0.001)	

Kendall’s Coefficient of Concordance (W) measures expert agreement across multiple statements. Statistically significant agreement was measured using p < 0.001

Regarding research priorities, the highest ranked statements suggested the investment in development of biomarkers and exploration of long-term outcomes and quality of life for leukemia survivors. Other high-ranked statements suggested factors such as development of novel therapies, factors affecting treatment adherence, mechanisms of drug resistance, and evaluations of the cost-effectiveness of novel therapies in comparison to traditional chemotherapy.

### Discussion

The aim of this study was to elicit consensus from healthcare experts in order to identify and prioritize key areas for improving leukemia care within the South African healthcare system. To our knowledge, this is the first study to evaluate the healthcare professionals’ opinions on priority setting in leukemia care and services.

The application of the modified Delphi technique provided a structured approach to systematically identify priority statements and rank improvement and research priorities. From statement ratings in both rounds and ranking in round 2, there was consensus around the type of statements considered as most important or of highest priority. Our study identified 67 priority statements within 6 themes considered by experts as priorities for diagnosis, treatments, management, access to care, resource allocation, and quality of life for leukemia. Across these themes, the emerged consensus on several key priorities reflected the shared perspectives of the expert participants. A total of 24 statements reached a high consensus threshold (≥ 90% agreement), reflecting broad expert agreement on priority areas for leukemia care.

Among the key findings, five major areas emerged with significant implications for improving care in South Africa. The primary finding was the accessibility of timely and accurate diagnostic techniques, this has been identified as critical importance in the management of leukemia globally [45]. Basic diagnostic tests such as complete blood counts and differential counts were identified as an essential initial step in leukemia diagnosis [36, 37]. However, more advanced diagnostic methods such as Fluorescence in situ hybridization (FISH) or Polymerase Chain Reaction (PCR) for molecular testing and flow cytometry for immunophenotyping were emphasized as crucial for the classification of leukemia subtypes and aid in prognosis and treatment planning, which is aligned with international guidelines and recommendations [35, 36]. These advanced methods are currently limited in availability across many regions of South Africa, presenting a significant barrier to cancer care. Diagnostic accuracy directly influences prognosis, treatment response assessment, and progression monitoring [45]. Furthermore, cancer treatment outcomes are also largely determined by accurate and timely diagnosis [46]. Enhancing access to these diagnostic tools is a key area for policy and service development, particularly in South Africa.

Another key priority was the need for treatment planning and risk stratification. In terms of treatment, experts emphasized the need for a balanced approach that includes efficacy, risk stratification, and evaluation of comorbidities and resource limitations. While chemotherapy remains a cornerstone for treating acute leukemia, with the options of combination therapies in eligible patients [35], there is growing recognition of the role of newer therapies, such as targeted therapies and immunotherapies, particularly for chronic leukemia. Effective treatment planning must be individualized, factoring in these considerations to improve patient outcomes. The availability of these treatments and their integration into clinical practice in South Africa will require ongoing investment and training to ensure their effectiveness and accessibility [7, 36].

Moreover, effective leukemia management extends beyond initial treatment and includes follow-up monitoring, symptom management, addressing adverse effects, and supportive care measures [47]. Follow-up monitoring and symptom management are critical for managing leukemia beyond the initial treatment phase. The need for standardized treatment-related complications and post-treatment guidelines was identified as a priority. This includes managing common hematological symptoms such as anemia, nausea, and mucositis, which can significantly impact patient quality of life (QoL). Supportive care measures such as pain management, psychological support, and infection prevention were also deemed a priority. Managing cancer symptoms (e.g., anemia, nausea, or mucositis) and providing supportive care measures (e.g., pain management, blood transfusions, or nutritional support) have been found to play an important role in improving treatment outcomes and improving QoL [47, 48]. Similar recommendations have been made in other studies, emphasizing the role of holistic care in improving both treatment outcomes and QoL [4, 47–49].

The study also highlighted the formation of multidisciplinary teams, which was highlighted as an essential component of improving leukemia care. Evidence indicates that a team approach to cancer care, where health professionals together consider all options and develop an individual treatment plan, can improve survival and quality of life [46, 50]. This approach also addresses the complex needs of leukemia patients, encompassing medical, psychological, and social dimensions of care. The establishment of multidisciplinary teams, particularly in resource-limited settings, will require adequate workforce training and investment in healthcare infrastructure [47].

While biobanks were ranked lower in terms of immediate priority, participants identified their potential long-term value in leukemia management. Biobanks facilitate the storage of bone marrow and blood samples for active monitoring, enabling more personalized and precise treatment regimens [37]. A study focused on prioritizing evidence-based health strategies found that lower priority rating in consensus studies on health measures may be an indication of perceived barriers or resource availability among participants [43]. This is evident as participants noted that South Africa does not have any established biobanks for leukemia and questioned the feasibility of establishing biobanks in the near future.

Despite the perceived barriers to establishing biobanks in South Africa, their importance for research and clinical monitoring cannot be overlooked. Future research should explore the feasibility and potential benefits of setting up biobanks in South Africa and other LMICs.

These five priority areas reflect the complex interplay of diagnostic, treatment, and supportive care needs in leukemia management. They also underscore the challenges faced by healthcare professionals in resource-constrained environments, where limited access to advanced technologies, therapies, and skilled personnel presents barriers to optimal care. Addressing these priorities will require concerted efforts from policymakers, healthcare providers, and researchers to enhance both the accessibility and quality of leukemia care in South Africa.

Additionally, the ranking of improvement areas and research priorities provided valuable insights into the future directions for leukemia care in South Africa. Top improvement areas included enhancing diagnostic capabilities [46], increasing access to advanced treatments [51], and improving patient education and support services [48]. Research priorities focused on developing cost-effective diagnostic and treatment methods [52], understanding the genetic and environmental factors influencing leukemia in the South African population [53], and evaluating the long-term outcomes of different treatment strategies [54].

Overall, this study was able to identify specific priorities that provide both high impact and modifiable improvements if implemented or established to reduce disparities and improve patient outcomes. These priorities address both implementation gaps (requiring improvements and/or service re-organization) and evidence gaps (requiring further research) and reflect the perspectives of healthcare professionals who are considered the end-users of priority research. The improvement and research priorities identified in this study provide an important framework from which to develop future research projects.

### Strengths and limitations

A significant strength of this study was the expert participants, who had a wide range of experience from different geographic regions in South Africa and selected to best represent the breadth of knowledge and clinical expertise in the field of leukemia. This diverse group of experts allowed for a broader perspective (covering different patient profiles) and generalization of consensus to be achieved. The modified Delphi method provided a systematic means of gaining consensus from the variety of experts. The online format enabled inclusion of participants from geographically dispersed locations within South Africa. An additional advantage was the anonymity of the Delphi survey rounds that meant no one voice was given precedence and participants had time to consider their responses.

Nevertheless, this study was not without limitations. Priority statements were mainly generated and then considered by participants with experience and research within the South African healthcare setting, therefore the results only reflect questions from a South African standpoint. Another key limitation was the number of Clinical Hematologists included in the study; however, this reflects the real-world shortage of Clinical Hematologists in South Africa. Despite the limited number, all participants were specialists actively involved in leukemia care, making their input highly relevant. Furthermore, the standard of care in a particular healthcare setting varies in different countries with different healthcare systems, which would make incorporating an international perspective difficult. It is important to recognize that some of our findings and key statements are likely to have some relevance to other countries. However, our priorities may require adaption or be applied cautiously to other healthcare systems, which may represent a scope for future research. Additionally, it is important to recognize that Delphi studies results are considered a ‘group consensus’ and not necessarily deemed to be ‘best,’ ‘correct,’ or ‘expert’ results [25]. Lastly, while the study focused on expert opinions, future research should incorporate patient perspectives to ensure that identified priorities align with patient needs and experiences.

## Conclusion

Evidence of key aspects required for leukemia care and services are essential for national healthcare systems to inform policy-making, resource allocation, and service improvements. This study identified key priorities for leukemia care within the South African healthcare system, providing an evidence-based framework through expert consensus. This study identified critical priorities for leukemia care within the South African healthcare system, providing a structured, expert-driven framework for decision-making. Through the Delphi methodology, expert consensus was obtained on essential indicators related to leukemia diagnosis, treatment strategies, healthcare access, and quality of life considerations. Furthermore, consensus was reached on key areas for improvement, including improved access to care, optimized resource allocation, and enhanced service delivery.

These findings offer a foundation for further research, encouraging evidence-based discussions around priority setting in leukemia care. Additional research is needed to validate these findings using multi-criterion decision techniques and observational methods.

## Supplementary Information

Below is the link to the electronic supplementary material.

Supplementary file1 (DOCX 119 KB)

## Data Availability

Data are provided within the manuscript and supplementary information file.

## Abbreviations

ASH: American society of hematology
CREDES: Conducting and reporting of Delphi studies
CR: Complete remission
EHA: European hematology association
ELN: European LeukemiaNet
FISH: Fluorescence in situ hybridization
GBD: Global burden of disease
HCPs: Healthcare professionals
LMICs: Low- and middle-income countries
MRD: Measurable residual disease
NCCN: National comprehensive cancer network
NICE: National institute of health and care excellence
PCR: Polymerase chain reaction
QoL: Quality of life
SD: Standard deviation
SDGs: Sustainable development goals
