# Supplementary material for: Priority setting for improved leukemia management and research in South Africa: a modified Delphi study
Source: Cancer Causes Control. 2025 Mar 4;36(8):781–93. doi: 10.1007/s10552-025-01979-4 (PMC12289788; doi:10.1007/s10552-025-01979-4)
Supplement: Supplementary file 1 — Supplementary file1 (DOCX 119 KB) [file 10552_2025_1979_MOESM1_ESM.docx]

**Cancer Causes & Control**

Priority Setting for Leukemia Management, Improvement, and Research in South Africa: a modified Delphi study

**SUPPLEMENTARY MATERIAL**

**Authors**

1) Rochelle Woudberg, MSc - **Corresponding Author**

[SMTROC001@myuct.ac.za](mailto:SMTROC001@myuct.ac.za)

Health Economics Unit, School of Public Health, University of Cape Town, South Africa

2) Edina Sinanovic, PhD

Health Economics Unit, School of Public Health, University of Cape Town, South Africa

# S1: Literature keyword search strategy

| **Table 1:** Search strategy of PubMed and Scopus databases. | | |
| --- | --- | --- |
| **Database** | **Search terms** | **Results** |
| PubMed | (((acute leukaemia[Title/Abstract] OR acute leukemia[Title/Abstract]) OR (acute lymphoblastic leukemia[Title/Abstract] OR acute lymphocytic leukemia[Title/Abstract]) OR (acute myeloid leukemia[Title/Abstract])) OR ((chronic leukaemia[Title/Abstract] OR chronic leukemia[Title/Abstract]) OR (chronic myeloid leukemia[Title/Abstract] OR chronic lymphocytic leukemia[Title/Abstract]))) AND ((diagnosis[Mesh]) OR (treatment[Mesh]) OR (management[Mesh])) AND ((“priority setting” OR priorities OR priority OR “resource allocation”) OR guidelines)) | 540 |
| Scopus | (( TITLE-ABS-KEY ( “acute leukemia” ) OR TITLE-ABS-KEY ( "chronic leukemia" ) AND TITLE-ABS-KEY ( diagnosis ) OR TITLE-ABS-KEY ( treatment ) OR TITLE-ABS-KEY ( management ) AND TITLE-ABS-KEY ( guidelines ) OR TITLE-ABS-KEY ( “priority setting” )) | 1013 |

**Details of currently available high-quality evidence:**

Published in the last 5 years (2019 onwards), in English

Databases searched using a targeted title/abstract search and variety of search terms to reflect the concepts or specifics of each individual indicative question (see table 1)

Databases:

- PubMed (Medline)
- Scopus

Additional Societies and guidelines:

- American Society of Hematology (ASH)
- The European Hematology Association (EHA)
- National Institute for Health and Care Excellence (NICE) guidelines

# S2: Literature Review Results Flowchart

**
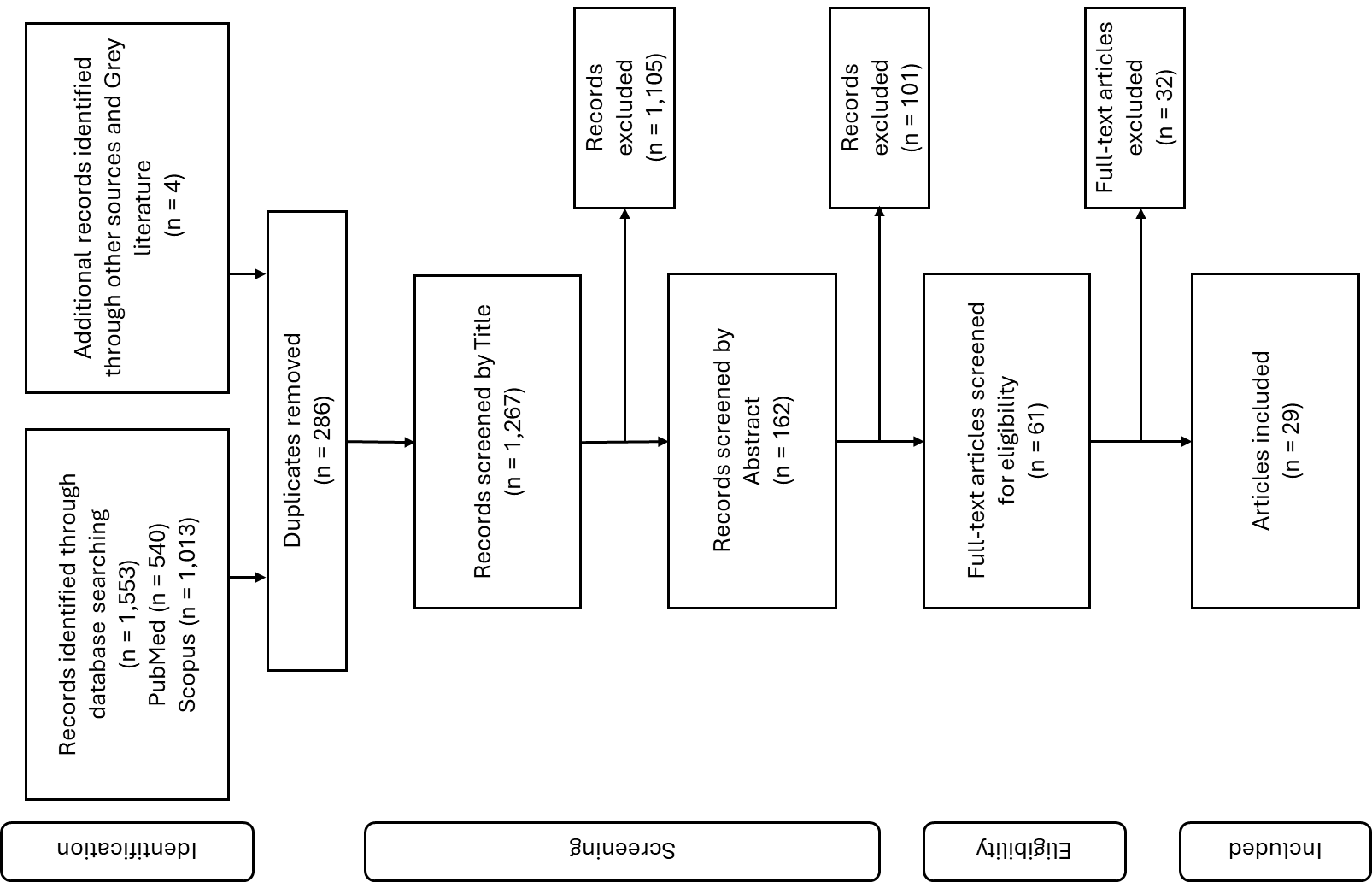
**

**Figure 1. Flow chart showing method of literature search and results of literature review**

# S3: Characteristics of included literature

Characteristics of included articles and guidelines on leukemia care and services for prioritization

| **Author, year** | **Title** |
| --- | --- |
| Agrwal and Sahi (2020) | National Comprehensive Cancer Network Guidelines for Pediatric Acute Lymphoblastic Leukemia - UPDATE |
| Andersson *et al.* (2022) | A survey on thromboprophylaxis and coagulation assessment in children and young adults with acute lymphoblastic leukaemia (ALL) in the Nordic and Baltic countries: Different practices of assessment and management |
| Arat *et al.* (2023) | Unmet clinical needs and challenges in the management of acute leukemia in Turkey: a modified Delphi study |
| Blackmon and Hourigan (2023) | Test Then Erase? Current Status and Future Opportunities for Measurable Residual Disease Testing in Acute Myeloid Leukemia |
| Boyiadzis *et al.* (2020) | The Society for Immunotherapy of Cancer (SITC) clinical practice guideline on immunotherapy for the treatment of acute leukemia |
| Brown *et al.* (2021) | Acute lymphoblastic leukemia, version 2.2021, NCCN Clinical Practice Guidelines in Oncology |
| Brown *et al.* (2020) | Pediatric Acute Lymphoblastic Leukemia, Version 2.2020, NCCN Clinical Practice Guidelines in Oncology |
| Chea *et al.* (2024) | Minimal Residual Disease in Acute Myeloid Leukemia: Old and New Concepts |
| Deinigner *et al.* (2020) | Chronic Myeloid Leukemia, Version 2.2021, NCCN Clinical Practice Guidelines in Oncology |
| DeWolf and Tallman (2020) | How I treat relapsed or refractory AML |
| Ding *et al.* (2022) | Factors that contribute to disparities in time to acute leukemia diagnosis in young people: an in-depth qualitative interview study |
| Dohner *et al.* (2022) | Diagnosis and management of AML in adults: 2022 recommendations from an international expert panel on behalf of the ELN |
| Eichhorst *et al.* (2021) | Chronic lymphocytic leukaemia: ESMO Clinical Practice Guidelines for diagnosis, treatment, and follow-up |
| Goswami *et al.* (2020) | Quality-of-life issues and symptoms reported by patients living with haematological malignancy: a qualitative study |
| Granroth *et al.* (2022) | Progress and Challenges in Survivorship After Acute Myeloid Leukemia in Adults |
| Guijarro *et al.* (2023) | Novel Tools for Diagnosis and Monitoring of AML |
| Hampel and Parikh (2022) | Chronic lymphocytic leukemia treatment algorithm 2022 |
| Heuser *et al.* (2021) | Update on MRD in acute myeloid leukemia: a consensus document from the European LeukemiaNet MRD Working Party |
| Hochhaus *et al.* (2020) | European LeukemiaNet 2020 recommendations for treating chronic myeloid leukemia |
| Jabbour and Kantarjian (2022) | Chronic myeloid leukemia: 2022 update on diagnosis, therapy, and monitoring |
| Jie *et al.* (2020) | Unmet supportive care needs and its relation to quality of life among adult acute leukaemia patients in China: a cross-sectional study |
| Kale *et al.* (2021) | Old drugs, new uses: Drug repurposing in hematological malignancies |
| Mathews *et al.* (2023) | Management of B-cell lineage acute lymphoblastic leukemia: expert opinion from an Indian panel via Delphi consensus method |
| Meillon-Garcia and Demichelis-Gomez (2020) | Access to Therapy for Acute Myeloid Leukemia in the Developing World: Barriers and Solutions |
| Rani *et al.* (2020) | Utility of immunohistochemistry on bone marrow trephine biopsy for the diagnosis and classification of acute leukemia |
| Salas *et al.* (2022) | Intensive end-of-life care in acute leukemia from a French national hospital database study (2017–2018) |
| Short *et al.* (2019) | Recommendations for the Assessment and Management of Measurable Residual Disease in Adults with Acute Lymphoblastic Leukemia: A Consensus of North American Experts |
| Valli *et al.* (2020) | Has Drug Repurposing Fulfilled Its Promise in Acute Myeloid Leukaemia? |
| Wang *et al.* (2020) | Management of hemostatic complications in acute leukemia: guidance from the SSC of the ISTH |

# S4: Questionnaire list of priority statements

Proposed priority statement generation list for Delphi study from literature review in Round 1

(Arranged according to themes)

|  | **STATEMENT** |
| --- | --- |
| **DIAGNOSIS** | |
| 1. | Medical history and Physical examination at initial examination |
| 2. | Complete blood counts and Differential counts for the identification of changes in blood components |
| 3. | Bone marrow aspiration for assessment of cell morphology |
| 4. | Bone Marrow Trephine Biopsy for assessment of cellularity and pattern of blast cell infiltration |
| 5. | Cytogenetic analysis for the identification of chromosomal abnormalities associated with specific leukemia subtypes |
| 6. | Molecular tests (e.g., FISH, PCR &/or NGS) for the detection of specific genetic abnormalities |
| 7. | FISH as an effective technique for molecular testing |
| 8. | PCR as an effective technique for molecular testing |
| 9. | Next-generation sequencing (NGS) as an effective technique for molecular testing |
| 10. | Immunohistochemistry for classification of leukemia |
| 11. | Immunophenotyping for the identification of cell surface markers and antigens |
| 12. | Flow cytometry as an effective technique for Immunophenotyping |
| 13. | Immunohistochemistry in the absence of flow cytometry or in remote areas for Immunophenotyping |
|  | **Additional tests performed during initial diagnosis:** |
| 14. | Blood chemistry panel |
| 15. | Liver function tests |
| 16. | Coagulation panel |
| 17. | Tumour lysis syndrome panel |
| 18. | Hepatitis B/C, HIV, CMV, EBV |
| 19. | Evaluated for opportunistic infections (as appropriate) |
| 20. | Lumbar puncture (on indication) |
| 21. | Initial imaging in patients with newly diagnosed leukemia |
| 22. | CT (on indication) |
| 23. | MRI (on indication) |
| 24. | PET (on indication) |
| 25. | *****Chest X ray (on indication) |
|  | **Additional diagnostic considerations:** |
| 26. | Routine tracking of immune competence of patients |
| 27. | HLA testing in patients with newly diagnosed leukemia |
| 28. | Performing a Donor search |
| 29. | Pregnancy testing in females of childbearing age prior to initiating treatment |
| 30. | Conducting evaluation for testicular involvement in males when indicated |
| 31. | Fertility counselling |
| 32. | Cardiac function testing to monitor potential treatment-related cardiotoxicity |
| 33. | Biobanking patient samples (e.g., for long-term monitoring or future research) |
| **TREATMENT** | |
|  | ***Treatment options in Acute Lymphocytic Leukaemia:*** |
| 34. | Chemotherapy as the primary ALL treatment |
| 35. | Targeted therapy for eligible patients |
| 36. | Immunotherapy for eligible patients |
| 37. | Radiation therapy for CNS involvement |
| 38. | Stem cell / Bone marrow transplantation in relapsed disease |
| 39. | Clinical trials for eligible patients / relapsed disease |
| 40. | Combination treatment protocol specifically for Philadelphia chromosome-positive ALL |
|  | ***Treatment options in Acute Myeloid Leukaemia:*** |
| 41. | Chemotherapy as the primary induction AML treatment |
| 42. | Targeted therapy for eligible patients |
| 43. | Immunotherapy for eligible patients |
| 44. | Radiation therapy for CNS involvement |
| 45. | Stem cell or Bone marrow transplantation in relapse or high-risk patients |
| 46. | Clinical trials for eligible patients / relapsed disease |
| 47. | Combination treatment protocol for enhanced AML treatment efficacy |
|  | ***Treatment options in Chronic Lymphocytic Leukaemia:*** |
| 48. | Chemotherapy for eligible patients |
| 49. | Targeted therapy for enhanced efficacy in CLL |
| 50. | Immunotherapy in relapse or high-risk patients |
| 51. | Radiation therapy on indication |
| 52. | Stem cell or Bone marrow transplantation for eligible patients |
| 53. | Clinical trials for eligible patients / relapsed disease |
| 54. | Combination treatment protocol for enhanced CLL treatment efficacy |
|  | ***Treatment options in Chronic Myeloid Leukaemia:*** |
| 55. | Chemotherapy for eligible patients |
| 56. | Targeted therapy for enhanced efficacy |
| 57. | Immunotherapy in relapse or high-risk patients |
| 58. | Radiation therapy on indication |
| 59. | Stem cell or Bone marrow transplantation for eligible patients |
| 60. | Clinical trials for eligible patients / relapsed disease |
| 61. | Combination treatment protocol for enhanced CML treatment efficacy |
|  | ***Factors in determining treatment strategies:*** |
| 62. | Age consideration to balance efficacy and tolerance |
| 63. | Risk stratification for tailoring treatment plans |
| 64. | Evaluation of comorbidities to adapt treatment protocols |
| 65. | Financial constraints consideration for patients in the public sector |
|  | ***Measurable residual disease monitoring:*** |
| 66. | Routine measurable residual disease (MRD) monitoring in leukaemia patients |
| 67. | Flow cytometry in monitoring MRD |
| 68. | Use of PCR if available for MRD monitoring |
| 69. | Next-generation sequencing if available for MRD monitoring |
|  | ***Additional treatment considerations:*** |
| 70. | Adoption of risk-stratification in Leukaemia treatment |
| 71. | Combination therapies (e.g., chemo & immunotherapy) for improved treatment outcomes |
| 72. | Extended maintenance or continuation therapy to prevent disease relapse after post remission induction and consolidation therapy |
| 73. | Personalised medicine approaches (e.g., based on genetic profiling) in tailoring treatment |
| 74. | Treatment strategies specifically tailored for paediatric leukaemia patients |
| 75. | Use of specific paediatric treatment regimens, for adolescent and young adult patients |
| 76. | Utilizing patient MRD status for treatment decisions |
| 77. | Novel treatment options in managing refractory or relapsed leukaemia |
| 78. | Investing in development of novel combination therapies |
| 79. | Exploring the repurposing of treatments for leukaemia |
| 80. | Prioritising research efforts aimed at overcoming treatment resistance |
| 81. | Development of standardized treatment guidelines for the different types of leukaemia |
| 82. | Education for Healthcare Professionals about emerging treatment paradigms and guidelines |
| **MANAGEMENT** | |
|  | ***Follow up & monitoring:*** |
| 83. | Routine blood tests for monitoring patients' response to treatment and detecting early signs of relapse |
| 84. | PROs in follow-up visits to assess symptoms, quality of life, and treatment-related adverse effects |
| 85. | Minimizing treatment-related toxicities and long-term adverse effects |
| 86. | Long-term monitoring for late effects (e.g., secondary malignancies, cardiotoxicity) in survivors |
| 87. | Developing early intervention strategies for managing treatment-related complications |
| 88. | Survivorship care plans for post-treatment, outlining follow-up schedules, potential late effects, and health maintenance recommendations |
| 89. | Telemedicine and remote monitoring technologies in follow-up care |
| 90. | Guidelines for treatment-related complications and post-treatment follow-up |
| 91. | *Development and implementation of supportive care guidelines |
|  | ***Symptoms and Adverse effects:*** |
| 92. | Haematological symptoms (e.g., anaemia, thrombocytopaenia) management to improve treatment tolerance and prevent complications |
| 93. | Nausea and vomiting to improve treatment adherence |
| 94. | Fatigue and malaise |
| 95. | Diarrhea and constipation |
| 96. | Low appetite and weight loss |
| 97. | Mucositis and oral complications for effective pain management |
| 98. | Peripheral neuropathy |
|  | ***Supportive Care Measures:*** |
| 99. | Pain management to improve quality of life |
| 100. | Nutritional support for improving treatment response and preserve functional status |
| 101. | Psychological support to improve overall patient management |
| 102. | Blood transfusions as a supportive care measure to improve patient tolerance to aggressive therapies |
| 103. | Infection prevention to reduce treatment-related morbidity |
| 104. | Thrombosis treatment in adults |
|  | ***Patient education:*** |
| 105. | Providing educational materials for newly diagnosed leukemia patients about their diagnosis and treatment options |
| 106. | Education about potential adverse effects and given clear instructions for call parameters for any toxicities to treatments |
| 107. | Educating leukemia survivors about the signs and symptoms of disease recurrence |
| 108. | Education on available support services and resources (e.g., patient support groups, symptom management, financial assistance programs) |
| 109. | Providing education material and communications that meet diverse needs and preferences of leukaemia patients (e.g., language, literacy level, cultural background) |
| **RESOURCE ALLOCATION** | |
| 110. | Availability of affordable and accessible basic diagnostic technologies |
| 111. | Availability of standard chemotherapy for the different types of leukaemia |
| 112. | Availability of diagnostic infrastructure for specialized tests (e.g., flow cytometry, genetic analysis, molecular testing) |
| 113. | Availability of resources to manage treatment-related adverse events |
| 114. | Improving healthcare infrastructure in underserved or remote areas |
| **ACCESS TO CARE** | |
| 115. | Ensuring equitable access to specialist diagnostic resources, services, equipment, and supplies (e.g., imaging, haematology, pathology, chemotherapy, radiation oncology) |
| 116. | Timely delivery of diagnosis and treatments |
| 117. | Adequate healthcare workforce and service providers |
| 118. | Multidisciplinary teams in oncology therapy sites (e.g., haematologists, clinical nurses, clinical pharmacists, laboratory specialists) |
| 119. | Adequate guidelines on patient referrals and management |
| 120. | Equitable access to novel and costly leukaemia treatment options |
| 121. | Access to follow-up care and monitoring services, including those in underserved or remote areas |
| 122. | *****Building relationships with staff in peripheral hospitals to improve the overall quality of care |
| **QUALITY OF LIFE (QoL)** | |
| 123. | Considering long-term effects and survivorship issues when prioritizing leukaemia treatment approaches |
| 124. | Providing supportive care interventions to improve patient outcomes and overall quality of life |
| 125. | Addressing emotional well-being (e.g., anxiety, depression) as part of patient care |
| 126. | Assessing potential financial burden in treatment scheduling and options for patients |
| 127. | Early integration of palliative care services in leukaemia treatment pathway |
| 128. | *****Providing support for siblings and parents of leukaemia patients to improves the patient's care experience |

**Abbreviations:** ALL, Acute Lymphocytic Leukaemia; AML, Acute Myeloid Leukaemia; CLL, Chronic Lymphocytic Leukaemia; CML, Chronic Myeloid Leukaemia; FISH, Fluorescence in situ hybridization; MRD, Measurable residual disease; NGS, Next-generation sequencing; PCR, Polymerase chain reaction; PROs, Patient-reported outcomes; QoL, Quality of life.

*****Additional statements suggested by participants in round 1.

# S5: Expert consensus results for priority statements, Delphi Round 1 & 2

Full list of all priority statements and their mean rating and level of consensus: Delphi Round 1 & 2 results combined.

|  |  | **Round 1 (n = 14)** | | **Round 2 (n = 10)** | | **Consensus Achieved** |
| --- | --- | --- | --- | --- | --- | --- |
| **Priority Statement** |  | **Mean (SD)*** | **% agreement** | **Mean (SD)*** | **% agreement** |  |
| **Diagnosis** |  |  |  |  |  |  |
| Medical history and Physical examination at initial examination |  | 4.86 (0.36) | **86%** |  |  | **Yes** |
| Complete blood counts and Differential counts for the identification of changes in blood components |  | 5.00 (0.00) | **100%** |  |  | **Yes** |
| Bone marrow aspiration for assessment of cell morphology |  | 4.86 (0.36) | **86%** |  |  | **Yes** |
| Bone Marrow Trephine Biopsy for assessment of cellularity and pattern of blast cell infiltration |  | 4.43 (1.09) | 69% | 3.90 (1.20) | 30% | No |
| Cytogenetic analysis for the identification of chromosomal abnormalities associated with specific leukemia subtypes |  | 4.86 (0.36) | **86%** |  |  | **Yes** |
| Molecular tests (e.g., FISH, PCR &/or NGS) for the detection of specific genetic abnormalities |  | 5.00 (0.00) | **100%** |  |  | **Yes** |
| FISH as an effective technique for molecular testing |  | 4.71 (0.47) | 71% | 4.70 (0.95) | **90%** | **Yes** |
| PCR as an effective technique for molecular testing |  | 4.57 (0.51) | 57% | 4.20 (0.92) | 40% | No |
| Next-generation sequencing (NGS) as an effective technique for molecular testing |  | 4.57 (0.51) | 57% | 3.80 (1.14) | 30% | No |
| Immunohistochemistry for classification of leukemia |  | 4.43 (0.83) | 57% | 3.90 (1.37) | 40% | No |
| Immunophenotyping for the identification of cell surface markers and antigens |  | 5.00 (0.00) | **100%** |  |  | **Yes** |
| Flow cytometry as an effective technique for Immunophenotyping |  | 4.93 (0.27) | **93%** |  |  | **Yes** |
| Immunohistochemistry in the absence of flow cytometry or in remote areas for Immunophenotyping |  | 3.79 (1.53) | 42% | 4.20 (1.14) | 60% | No |
| ***Additional tests performed during initial diagnosis:*** |  |  |  |  |  |  |
| Blood chemistry panel |  | 4.29 (1.33) | 62% | 4.50 (0.97) | 70% | No |
| Liver function tests |  | 3.93 (1.27) | 31% | 4.30 (1.06) | 60% | No |
| Coagulation panel |  | 4.43 (0.65) | 50% | 4.40 (0.97) | 60% | No |
| Tumour lysis syndrome panel |  | 4.64 (0.50) | 64% | 4.20 (1.23) | 60% | No |
| Hepatitis B/C, HIV, CMV, EBV |  | 4.36 (0.63) | 43% | 4.30 (0.95) | 50% | No |
| Evaluated for opportunistic infections (as appropriate) |  | 4.43 (0.65) | 50% | 3.90 (1.37) | 40% | No |
| Lumbar puncture (on indication) |  | 4.36 (0.93) | 57% | 4.30 (1.25) | 60% | No |
| Initial imaging in patients with newly diagnosed leukemia |  | 4.07 (1.00) | 36% | 3.56 (1.01) | 11% | No |
| CT (on indication) |  | 3.07 (1.49) | 17% | 3.60 (1.26) | 20% | No |
| MRI (on indication) |  | 2.79 (1.42) | 9% | 3.30 (1.25) | 20% | No |
| PET (on indication) |  | 2.79 (1.48) | 18% | 2.90 (1.37) | 10% | No |
| *****Chest X ray (on indication) |  | N/A | N/A | 4.20 (1.14) | 60% | No |
| ***Additional diagnostic considerations:*** |  |  |  |  |  |  |
| Routine tracking of immune competence of patients |  | 3.71 (1.38) | 33% | 3.80 (1.03) | 20% | No |
| HLA testing in patients with newly diagnosed leukemia |  | 3.29 (1.38) | 25% | 4.20 (1.23) | 50% | No |
| Performing a Donor search |  | 3.14 (1.46) | 27% | 4.30 (1.06) | 60% | No |
| Pregnancy testing in females of childbearing age prior to initiating treatment |  | 4.50 (0.94) | 71% | 4.67 (1.00) | **89%** | **Yes** |
| Conducting evaluation for testicular involvement in males when indicated |  | 4.43 (0.94) | 64% | 4.60 (0.97) | **80%** | **Yes** |
| Fertility counselling |  | 4.43 (0.85) | 57% | 4.30 (0.95) | 50% | No |
| Cardiac function testing to monitor potential treatment-related cardiotoxicity |  | 4.64 (0.84) | **79%** |  |  | **Yes** |
| Biobanking patient samples (e.g., for long-term monitoring or future research) |  | 4.07 (1.00) | 43% | 4.00 (1.05) | 40% | No |
| **Treatment** |  |  |  |  |  |  |
| ***Treatment options in Acute Lymphocytic Leukaemia:*** |  |  |  |  |  |  |
| Chemotherapy as the primary ALL treatment |  | 4.79 (0.43) | **79%** |  |  | **Yes** |
| Targeted therapy for eligible patients |  | 4.14 (1.46) | 62% | 4.00 (1.15) | 40% | No |
| Immunotherapy for eligible patients |  | 4.36 (0.93) | 57% | 4.20 (0.92) | 40% | No |
| Radiation therapy for CNS involvement |  | 3.79 (1.53) | 42% | 4.30 (1.16) | 70% | No |
| Stem cell / Bone marrow transplantation in relapsed disease |  | 4.00 (1.57) | 58% | 4.50 (0.97) | 70% | No |
| Clinical trials for eligible patients / relapsed disease |  | 3.71 (1.68) | 42% | 4.50 (0.97) | 70% | No |
| Combination treatment protocol specifically for Philadelphia chromosome-positive ALL |  | 2.64 (2.44) | 43% | 4.60 (0.97) | **80%** | **Yes** |
| ***Treatment options in Acute Myeloid Leukaemia****:* |  |  |  |  |  |  |
| Chemotherapy as the primary induction AML treatment |  | 4.93 (0.27) | **93%** |  |  | **Yes** |
| Targeted therapy for enhanced efficacy in CLL |  | 4.00 (1.41) | 46% | 4.40 (0.97) | 60% | No |
| Immunotherapy in relapse or high-risk patients |  | 3.93 (1.21) | 38% | 3.90 (1.10) | 30% | No |
| Radiation therapy on indication |  | 2.86 (1.35) | 10% | 3.50 (1.58) | 40% | No |
| Stem cell or Bone marrow transplantation in relapse or high-risk AML patients |  | 4.86 (0.36) | **86%** |  |  | **Yes** |
| Clinical trials for eligible patients / relapsed disease |  | 4.50 (0.65) | 57% | 4.50 (0.97) | 70% | No |
| Combination treatment protocol for enhanced AML treatment efficacy |  | 2.71 (2.37) | 43% | 4.56 (1.01) | **78%** | **Yes** |
| ***Treatment options in Chronic Lymphocytic Leukaemia:*** |  |  |  |  |  |  |
| Chemotherapy for eligible patients |  | 3.21 (1.89) | 45% | 3.88 (1.25) | 38% | No |
| Targeted therapy for enhanced efficacy in CLL |  | 3.79 (1.85) | 67% | 4.50 (1.07) | **75%** | **Yes** |
| Immunotherapy in relapse or high-risk CLL patients |  | 3.64 (1.91) | 64% | 4.63 (1.06) | **88%** | **Yes** |
| Radiation therapy on indication |  | 1.86 (1.41) | 0% | 3.25 (1.39) | 13% | No |
| Stem cell or Bone marrow transplantation for eligible patients |  | 1.93 (1.44) | 14% | 2.75 (1.67) | 13% | No |
| Clinical trials for eligible patients / relapsed disease |  | 3.00 (1.88) | 27% | 4.25 (1.04) | 50% | No |
| Combination treatment protocol for enhanced CLL treatment efficacy |  | 1.43 (1.99) | 40% | 4.25 (1.04) | 50% | No |
| ***Treatment options in Chronic Myeloid Leukaemia:*** |  |  |  |  |  |  |
| Chemotherapy for eligible patients |  | 2.86 (1.70) | 30% | 3.50 (1.65) | 50% | No |
| Targeted therapy for enhanced efficacy in CML |  | 4.93 (0.27) | **93%** |  |  | **Yes** |
| Immunotherapy in relapse or high-risk patients |  | 2.36 (1.45) | 11% | 3.00 (1.41) | 20% | No |
| Radiation therapy on indication |  | 1.93 (1.21) | 0% | 2.90 (1.60) | 20% | No |
| Stem cell or Bone marrow transplantation for eligible patients |  | 3.07 (1.64) | 27% | 3.30 (1.49) | 30% | No |
| Clinical trials for eligible patients / relapsed disease |  | 4.29 (1.14) | 62% | 4.30 (1.06) | 60% | No |
| Combination treatment protocol for enhanced CML treatment efficacy |  | 2.07 (1.98) | 29% | 4.00 (1.00) | 33% | No |
| ***Factors in determining treatment strategies:*** |  |  |  |  |  |  |
| Age consideration to balance efficacy and tolerance |  | 4.71 (0.83) | **86%** |  |  | **Yes** |
| Risk stratification for tailoring treatment plans |  | 4.79 (0.80) | **93%** |  |  | **Yes** |
| Evaluation of comorbidities to adapt treatment protocols |  | 4.79 (0.80) | **93%** |  |  | **Yes** |
| Financial constraints consideration for patients in the public sector |  | 3.93 (1.69) | 67% | 4.70 (0.95) | **90%** | **Yes** |
| ***Measurable residual disease monitoring:*** |  |  |  |  |  |  |
| Routine measurable residual disease (MRD) monitoring in leukaemia patients |  | 4.50 (0.85) | 64% | 4.40 (0.97) | 60% | No |
| Flow cytometry in monitoring MRD |  | 4.93 (0.27) | **93%** |  |  | **Yes** |
| Use of PCR if available for MRD monitoring |  | 4.71 (0.47) | 71% | 4.70 (0.95) | **90%** | **Yes** |
| Next-generation sequencing if available for MRD monitoring |  | 3.71 (1.33) | 38% | 3.70 (1.06) | 30% | No |
| ***Additional treatment considerations:*** |  |  |  |  |  |  |
| Adoption of risk-stratification in Leukaemia treatment |  | 4.71 (0.83) | **86%** |  |  | **Yes** |
| Combination therapies (e.g., chemo & immunotherapy) for improved treatment outcomes |  | 4.71 (0.83) | **86%** |  |  | **Yes** |
| Extended maintenance or continuation therapy to prevent disease relapse after post remission induction and consolidation therapy |  | 3.29 (1.64) | 27% | 3.90 (0.99) | 30% | No |
| Personalised medicine approaches (e.g., based on genetic profiling) in tailoring treatment |  | 4.43 (0.85) | 57% | 4.10 (0.88) | 30% | No |
| Treatment strategies specifically tailored for paediatric leukaemia patients |  | 4.71 (0.83) | **86%** |  |  | **Yes** |
| Use of specific paediatric treatment regimens, for adolescent and young adult patients |  | 4.79 (0.80) | **93%** |  |  | **Yes** |
| Utilizing patient MRD status for treatment decisions |  | 4.79 (0.43) | **79%** |  |  | **Yes** |
| Novel treatment options in managing refractory or relapsed leukaemia |  | 4.50 (0.85) | 64% | 4.50 (0.97) | 70% | No |
| Investing in development of novel combination therapies |  | 4.50 (0.65) | 57% | 4.20 (1.23) | 60% | No |
| Exploring the repurposing of treatments for leukaemia |  | 3.79 (1.37) | 31% | 3.80 (1.40) | 40% | No |
| Prioritising research efforts aimed at overcoming treatment resistance |  | 4.71 (0.47) | 71% | 4.30 (1.25) | 70% | No |
| Development of standardized treatment guidelines for the different types of leukaemia |  | 4.93 (0.27) | **93%** |  |  | **Yes** |
| Education for Healthcare Professionals about emerging treatment paradigms and guidelines |  | 4.93 (0.27) | **93%** |  |  | **Yes** |
| **Management** |  |  |  |  |  |  |
| ***Follow up & monitoring:*** |  |  |  |  |  |  |
| Routine blood tests for monitoring patients' response to treatment and detecting early signs of relapse |  | 4.57 (1.09) | **85%** |  |  | **Yes** |
| PROs in follow-up visits to assess symptoms, quality of life, and treatment-related adverse effects |  | 4.71 (0.61) | **79%** |  |  | **Yes** |
| Minimizing treatment-related toxicities and long-term adverse effects |  | 4.71 (0.61) | **79%** |  |  | **Yes** |
| Long-term monitoring for late effects (e.g., secondary malignancies, cardiotoxicity) in survivors |  | 4.64 (0.63) | 71% | 4.50 (0.97) | 70% | No |
| Developing early intervention strategies for managing treatment-related complications |  | 4.71 (0.61) | **79%** |  |  | **Yes** |
| Survivorship care plans for post-treatment, outlining follow-up schedules, potential late effects, and health maintenance recommendations |  | 4.64 (0.63) | 71% | 4.40 (0.97) | 60% | No |
| Telemedicine and remote monitoring technologies in follow-up care |  | 3.50 (1.40) | 31% | 3.20 (1.23) | 10% | No |
| Guidelines for treatment-related complications and post-treatment follow-up |  | 4.79 (0.58) | **93%** |  |  | **Yes** |
| *****Development and implementation of supportive care guidelines |  | N/A | N/A | 4.40 (1.26) | 70% | No |
| ***Symptoms and Adverse effects:*** |  |  |  |  |  |  |
| Haematological symptoms (e.g., anaemia, thrombocytopaenia) management to improve treatment tolerance and prevent complications |  | 4.93 (0.27) | **93%** |  |  | **Yes** |
| Nausea and vomiting to improve treatment adherence |  | 4.64 (0.63) | 71% | 4.60 (0.97) | **80%** | **Yes** |
| Fatigue and malaise |  | 3.79 (1.42) | 38% | 4.00 (1.15) | 30% | No |
| Diarrhea and constipation |  | 4.29 (0.91) | 50% | 4.00 (1.15) | 40% | No |
| Low appetite and weight loss |  | 4.36 (0.93) | 57% | 4.20 (0.92) | 40% | No |
| Mucositis and oral complications for effective pain management |  | 4.71 (0.61) | **79%** |  |  | **Yes** |
| Peripheral neuropathy |  | 4.29 (1.38) | 69% | 4.40 (0.97) | 60% | No |
| ***Supportive Care Measures:*** |  |  |  |  |  |  |
| Pain management to improve quality of life |  | 5.00 (0.00) | **100%** |  |  | **Yes** |
| Nutritional support for improving treatment response and preserve functional status |  | 4.79 (0.43) | **79%** |  |  | **Yes** |
| Psychological support to improve overall patient management |  | 4.93 (0.27) | **93%** |  |  | **Yes** |
| Blood transfusions as a supportive care measure to improve patient tolerance to aggressive therapies |  | 4.86 (0.36) | **86%** |  |  | **Yes** |
| Infection prevention to reduce treatment-related morbidity |  | 4.93 (0.27) | **93%** |  |  | **Yes** |
| Thrombosis treatment in adults |  | 4.50 (0.65) | 57% | 4.38 (1.06) | 63% | No |
| ***Patient education:*** |  |  |  |  |  |  |
| Providing educational materials for newly diagnosed leukemia patients about their diagnosis and treatment options |  | 4.71 (0.83) | **86%** |  |  | **Yes** |
| Education about potential adverse effects and given clear instructions for call parameters for any toxicities to treatments |  | 4.86 (0.36) | **86%** |  |  | **Yes** |
| Educating leukemia survivors about the signs and symptoms of disease recurrence |  | 4.86 (0.36) | **86%** |  |  | **Yes** |
| Education on available support services and resources (e.g., patient support groups, symptom management, financial assistance programs) |  | 4.71 (0.47) | 71% | 4.44 (1.01) | 67% | No |
| Providing education material and communications that meet diverse needs and preferences of leukaemia patients (e.g., language, literacy level, cultural background) |  | 4.71 (0.47) | 71% | 4.56 (1.01) | **78%** | **Yes** |
| **Resource allocation** |  |  |  |  |  |  |
| Availability of affordable and accessible basic diagnostic technologies |  | 4.86 (0.36) | **86%** |  |  | **Yes** |
| Availability of standard chemotherapy for the different types of leukaemia |  | 4.86 (0.36) | **86%** |  |  | **Yes** |
| Availability of diagnostic infrastructure for specialized tests (e.g., flow cytometry, genetic analysis, molecular testing) |  | 4.86 (0.36) | **86%** |  |  | **Yes** |
| Availability of resources to manage treatment-related adverse events |  | 4.93 (0.27) | **93%** |  |  | **Yes** |
| Improving healthcare infrastructure in underserved or remote areas |  | 4.86 (0.36) | **86%** |  |  | **Yes** |
| **Access to care** |  |  |  |  |  |  |
| Ensuring equitable access to specialist diagnostic resources, services, equipment, and supplies (e.g., imaging, haematology, pathology, chemotherapy, radiation oncology) |  | 4.79 (0.43) | **79%** |  |  | **Yes** |
| Timely delivery of diagnosis and treatments |  | 4.79 (0.27) | **93%** |  |  | **Yes** |
| Adequate healthcare workforce and service providers |  | 5.00 (0.00) | **100%** |  |  | **Yes** |
| Multidisciplinary teams in oncology therapy sites (e.g., haematologists, clinical nurses, clinical pharmacists, laboratory specialists) |  | 4.57 (1.34) | **92%** |  |  | **Yes** |
| Adequate guidelines on patient referrals and management |  | 4.86 (036) | **86%** |  |  | **Yes** |
| Equitable access to novel and costly leukaemia treatment options |  | 4.43 (1.34) | 71% | 4.60 (0.97) | **80%** | **Yes** |
| Access to follow-up care and monitoring services, including those in underserved or remote areas |  | 4.86 (0.36) | **86%** |  |  | **Yes** |
| *****Building relationships with staff in peripheral hospitals to improve the overall quality of care |  | N/A | N/A | 4.60 (0.97) | **80%** | **Yes** |
| **Quality of life (QoL)** |  |  |  |  |  |  |
| Considering long-term effects and survivorship issues when prioritizing leukaemia treatment approaches |  | 4.71 (0.47) | 71% | 4.70 (0.95) | **90%** | **Yes** |
| Providing supportive care interventions to improve patient outcomes and overall quality of life |  | 4.79 (0.43) | **79%** |  |  | **Yes** |
| Addressing emotional well-being (e.g., anxiety, depression) as part of patient care |  | 4.79 (0.43) | **79%** |  |  | **Yes** |
| Assessing potential financial burden in treatment scheduling and options for patients |  | 4.71 (0.47) | 71% | 4.70 (0.95) | **90%** | **Yes** |
| Early integration of palliative care services in leukaemia treatment pathway |  | 4.86 (036) | **86%** |  |  | **Yes** |
| *Providing support for siblings and parents of leukaemia patients to improves the patient's care experience |  | N/A | N/A | 4.30 (1.25) | 70% | No |

SD = standard deviation

Abbreviations: ALL, Acute Lymphocytic Leukaemia; AML, Acute Myeloid Leukaemia; CLL, Chronic Lymphocytic Leukaemia; CML, Chronic Myeloid Leukaemia; FISH, Fluorescence in situ hybridization; MRD, Measurable residual disease; NGS, Next-generation sequencing; PCR, Polymerase chain reaction; PROs, Patient-reported outcomes; QoL, Quality of life.

Factors percentages that have reached consensus (rating of ≥75%), are shown in **bold**.

*Additional statements suggested by participants in round 1.
